# Multiple Social Identities Enhance Health Post-Retirement Because They Are a Basis for Giving Social Support

**DOI:** 10.3389/fpsyg.2016.01519

**Published:** 2016-10-17

**Authors:** Niklas K. Steffens, Jolanda Jetten, Catherine Haslam, Tegan Cruwys, S. Alexander Haslam

**Affiliations:** School of Psychology, The University of QueenslandBrisbane, QLD, Australia

**Keywords:** multiple identities, retirement, group membership, social identity, health, well-being, life satisfaction, social support

## Abstract

We examine the extent to which multiple social identities are associated with enhanced health and well-being in retirement because they provide a basis for giving and receiving social support. Results from a cross-sectional study show that retirees (*N* = 171) who had multiple social identities following (but not prior to) retirement report being (a) more satisfied with retirement, (b) in better health, and (c) more satisfied with life in general. Furthermore, mediation analyses revealed an indirect path from multiple social identities to greater satisfaction with retirement and better health through greater provision, but not receipt, of social support to others. These findings are the first to point to the value of multiple group membership post-retirement as a basis for increased opportunities to give meaningful support to others. We discuss the theoretical and practical implications for the management of multiple identities in the process of significant life transitions such as retirement.

## Introduction

Adjustment to retirement is a major challenge. Not least, this is because for many people retirement involves giving up a work identity with which they are highly engaged, and which provides them with a sense of respect and self-worth. In this context, it is not surprising that there is a lot of variation in people’s adjustment to retirement and that a substantial number of retirees report compromised health and well-being in the transition to retirement (Wang, 2007; Wang and Shi, 2014). Nevertheless, such findings beg the question of how best to manage the transition to retirement so as to allow people to maintain, and possibly enhance, their health and well-being. Speaking to this issue, the social identity approach suggests that individuals’ social group memberships — and the sense of ‘we’ and ‘us’ (or social identities) that they derive from these — are a basis for optimizing health and well-being (Haslam et al., 2009; Jetten et al., 2012; Tewari et al., 2012; Cruwys et al., 2014; Sani et al., 2015; Steffens et al., 2016c). Moreover, while a focus on social group memberships provides a useful theoretical framework for understanding why retirement puts people at risk (because it entails losing valued group memberships), it also points to potential ways to promote successful adjustment — namely, compensating for the loss of work group membership by strengthening memberships in other social groups post-retirement.

In the present research we draw on findings in the retirement, social identity, and social support literatures and propose that retirees’ multiple identities — that is, the number of social groups that they regard as self-defining — are associated with better adjustment and greater well-being. More specifically, we propose that having more (rather than few) social identities post-retirement can have benefits for health because these afford opportunities to make meaningful contributions to the lives of others through the provision of social support.

### Multiple Identities and Retirement Adjustment

Retirement constitutes a major life change and involves the loss of an activity that people have typically engaged in for a large part of their lives. Given the scale of the transition, it has the capacity to alter (for good or ill) how people live and feel (Pinquart and Schindler, 2007; Wang, 2007). To understand this process, scholars have focused on examining the factors that influence people’s ability to adjust to retirement (Coe and Zamarro, 2011; Wang and Shi, 2014). Specifically, evidence suggests that adjustment tends to be more successful where people engage in financial decision-making (Topa et al., 2009; Adams and Rau, 2011; Feldman and Beehr, 2011) and in planning the timing of their exit from work (Litwin, 2007; Brockmann et al., 2009; Wu et al., 2016), as well as participating in post-retirement leisure activities (Dorfman and Kolarik, 2005; Nimrod, 2007) and physical exercise (Lord et al., 2003; Nimrod et al., 2008). Yet despite considerable attention to these factors in retirement planning, a substantial number of people fail to adjust successfully (Wang, 2007). So there is clearly more to the story.

In addition to these factors, we propose that our capacity to adjust to retirement is also likely to be impacted by our *social* connectedness to others, and particularly to groups of others. Indeed, this proposition follows from the Social Identity Model of Identity Change (SIMIC; Jetten et al., 2009) which provides a way of conceptualizing the role that group memberships, and the social identities that underpin them, play in life changes such as retirement. This suggests that belonging to, and maintaining membership in, multiple social groups helps protect people’s health and well-being in the face of life transitions. In line with this general proposition, empirical evidence shows that belonging to multiple groups has health-related benefits for those experiencing a range of life-changing events such as starting life as a university student (Iyer et al., 2009), recovering from a stroke (Haslam et al., 2008), and adjusting to life following brain injury (Jones et al., 2011).

More directly relevant to our present concerns, there is evidence that membership of multiple social groups also reduces the risk of premature death in retirees. Speaking to this issue, Steffens et al. (2016a) investigated the contribution of social group ties to mortality in the 6 years following retirement in a nationally representative sample of over 400 individuals in England. One of the study’s most striking findings was that retirees who maintained multiple group ties during this transition were far less likely to die prematurely. More specifically, if people had two group memberships prior to retirement, they had only a 2% risk of early death if they maintained both, but this increased markedly if they lost one or both groups (to 5% and 12%, respectively). Importantly, these results controlled for factors that might otherwise be expected to restrict a person’s ability to maintain group memberships (e.g., physical health and age). Furthermore, the study also found that 6 years post-retirement life satisfaction was 10% lower for every group membership that people lost but did not replace.

Yet while this study demonstrates the importance of multiple social groups for people in retirement, it was not able to explore the contribution of self-identified groups to the observed relationships (i.e., those which are important and central to a person’s self-concept). This is relevant to the core tenet of self-categorization theory — that groups determine people’s psychology to the extent that they self-categorize and see themselves as part of a given group. Accordingly, attempting to index this construct through a list of pre-determined groups identified by researchers (rather than participants) will often fail to fully capture the diverse range of possible social groups to which people feel a psychological sense of belonging (for a more detailed discussion, see Cruwys et al., 2016). In particular, this approach may (a) miss groups that people see themselves as members of, but also (b) enhance the salience of a particular group by virtue of mentioning it, or (c) change the content and number of groups with which people identify in response to being primed with a list. It follows that any estimations based on pre-selected groups will tend to provide an inaccurate measure of multiple group membership and, ultimately, an inaccurate test of theoretical assertions related to this construct. In contrast, eliciting details of groups that participants generate themselves allows for a more accurate operationalization of the theoretical construct (than pre-determined groups) and, in turn, should provide a better index with which to test relationships of theoretical interest.

Even more important is the fact that this research was not able to examine the mechanisms that might underlie the beneficial effects of multiple identities. In particular, while there is strong evidence that social support is likely to be a critical mediator of the relationship between social identification and well-being (for a review, see Haslam et al., 2012), the role that this plays in helping retirees with multiple identities adjust to, and maintain their health in, retirement remains to be tested. The present research addresses both these limitations. In so doing it also simultaneously examines the degree to which both the provision and the receipt of support are implicated in the health-protecting benefits of multiple identities.

### Multiple Identities, Provision of Social Support, and Health

Theoretical and empirical research have suggested that self-categorization (and social identification) with a social group provides access to important sources of social support (Wiesenfeld et al., 2001; Levine et al., 2002, 2005; Reicher et al., 2006; Haslam et al., 2012). Added to this, research has demonstrated that this feeling of being socially supported is a key factor that explains (i.e., mediates) the effect that identification with a group has on people’s health. In this regard, there is abundant evidence of an indirect effect of social identification on health through received social support — both in groups in organizational contexts (van Dick et al., 2004; Haslam et al., 2005; Haslam and Reicher, 2006; McKimmie et al., 2009; Avanzi et al., 2015; Ketturat et al., 2016) as well as in leisure and other non-work group contexts (Branscombe et al., 1999; Crabtree et al., 2010). Yet, it has been suggested that if one group is beneficial for social support, then more groups are likely to further enhance the experience of social support. Indeed, the same pathway from identity to health via received social support has also been found in studies of multiple identities (Haslam et al., 2016b; Walter et al., 2016). From this plethora of studies, we can thus conclude that identification with (single and multiple) social groups is associated with better health and well-being in part because it provides people with a basis for being supported, and, more particularly, *perceiving* that they are supported, by others.

Yet while receiving support may be important in accounting for the link between social identity and health, another body of research indicates that it is often not the act of receiving but the act of *giving* support that is important in promoting health. Along these lines, evidence indicates that individuals’ health and well-being increase when they give support to others — be it in the form of (a) money (Dunn et al., 2008, 2010; Aknin et al., 2009, 2013), (b) time (Wheeler et al., 1998; Thoits and Hewitt, 2001; Campbell et al., 2009; Sarid et al., 2010; Choi and Kim, 2011), or (c) emotional help (Brown et al., 2003; Abolfathi Momtaz et al., 2014). Indeed, Brown et al.’s (2003) seminal study — which examined older married couples (all males were older than 65 years) over a period of 5 years — found that giving support to one’s spouse was more important in reducing mortality than receiving support from them (see also Abolfathi Momtaz et al., 2014). This then also suggests that changes in people’s lives that result in the loss of opportunities to give support (e.g., to engage in volunteering) can have negative health and well-being implications (e.g., by lowering people’s life satisfaction; Meier and Stutzer, 2008). Furthermore, it is noteworthy that the health-promoting effects of giving are particularly pronounced among older adults (Van Willigen, 2000; Musick and Wilson, 2003). For example, Greenfield and Marks (2004) found that the well-being of older adults who suffered from a loss of a role identity benefited to a great extent (and more than their younger counterparts) from volunteering their time to help others.

Drawing on the findings from these literatures, we argue that multiple identities in retirement are likely to be associated with enhanced health not only because they provide a basis for receiving support but also because they provide opportunities to *give* support. Indeed, of these two forms of social support, one might expect that the provision of social support will be especially protective in light of evidence that older adults derive greater health and well-being benefits from giving support than receiving it.

### The Present Research

In the present research, we investigate the link between multiple identities, retirement satisfaction, subjective quality of life, and subjective health in the transition to retirement. We hypothesize that multiple identities will be associated with retirees’ (a) better adjustment to the retirement transition (H1a), (b) better subjective health (H1b), and (c) greater subjective quality of life (H1c). We also examine these associations as they relate to people’s multiple identities before and after retirement in order to better understand the contribution of identities to health and well-being outcomes in the course of the transition. In line with findings by Steffens et al. (2016a) — who found that social group memberships following, but not prior, to retirement impacted mortality — we expect that multiple identities following retirement will be more important than those prior to retirement (H2). Furthermore, we examine the contribution that the nature of social support (i.e., giving and receiving) makes to the relationship between multiple identities and these three outcomes. In light of previous research, we hypothesize that not only receiving (H3) but also giving (H4) social support will mediate the effects of multiple identities on (a) retirement satisfaction, (b) subjective health, and (c) subjective quality of life.

## Materials and Methods

### Participants and Procedure

The study received ethics approval by the Behavioral and Social Sciences Ethical Review Committee at the first author’s university (Ref: 2012001231). We recruited a sample of 171 (71 female; 98 male; 2 undisclosed) retired individuals residing in Australia. Participants were recruited via different organizations that distributed an online survey to individuals who had recently retired (i.e., individuals who had stopped work and were not undertaking bridging or part-time work). This included the Ageing Mind Initiative’s [AMI] (2016) 50+ Registry, which has a database of people over 50 years who are willing to take part in research projects on ageing, and the Online Research Unit [ORU], 2016, the largest professional online research organization in Australia. Participants had an average age of 67.38 years (*SD* = 4.45) and they had been retired for an average of 3.63 years (*SD* = 3.86). Participants were invited to participate in a study titled “Transition to Retirement Survey.” After providing their informed consent, participants responded to the survey measures as indicated below. For all scales, unless stated otherwise, participants indicated their level of agreement with each item on scales ranging from 1 (*not at all*) to 7 (*completely*). Following completion of the survey, participants were debriefed and thanked for their involvement in the study.

### Measures

#### Multiple Identities

Participants used a multiple group membership listing task (Haslam et al., 2008) to identify the social groups that they belonged to and that they saw as important to their sense of self both pre- and post-retirement. Participants were given examples of categories and types of social groups (e.g., leisure or social groups, which could include book and gardening clubs; community groups, which could include church groups; workgroups which could include work teams), before being asked to provide up to a maximum of six social groups to which they belonged separately for the period before and after retirement. On average, participants reported being members of three groups prior to retirement (*M* = 2.99; *SD* = 1.89) and following retirement (*M* = 3.08; *SD* = 1.88). For each group that they identified, they also indicated on a scale ranging from 1 (*not important*) to 7 (*very important*) how important each group was to them.

#### Received Social Support

Participants responded to four items assessing their received social support (α = 0.92; adapted from Jetten et al., 2012; “People that are important to me help me with my tasks”; “People that are important to me provide me with emotional support”; “People that are important to me listen to me if I need to talk”; “People that are important to me make me feel loved and cared for”).

#### Provided Social Support

We adapted the same four items that were used to measure received social support to assess provided social support (α = 0.86; adapted from Jetten et al., 2012): “I help people that are important to me with their tasks,” “I provide emotional support to people that are important to me,” “I listen to those people that are important to me if they need to talk,” and “I make those people that are important to me feel loved and cared for.”

#### Satisfaction with Retirement

Participants responded to three items assessing their satisfaction with retirement, adapted from Spector’s (1997) Job Satisfaction Scale (α = 0.84). These items were: “In general, I am satisfied with being retired,” “In general, I don’t like being retired” [reverse-coded], and “In general, I like the fact that I am retired.”

#### Subjective Health Status

Participants responded to three items assessing their subjective health status (α = 0.97; adapted from Eriksson et al., 2001). These were: “At the moment, my health is very good,” “At the moment, my health is in an excellent condition,” and “At the moment, I feel very healthy.”

#### Subjective Quality of Life

Quality of life is a multi-dimensional construct that comprises both subjective and objective elements (Fernández-Ballesteros, 2011). In line with points made in the Introduction, in the present research we focused on subjective quality of life and used Diener et al. (1985) Satisfaction with Life Scale to assess this construct (α = 0.89). Items were as follows: “In most ways my life is close to ideal,” “The conditions of my life are excellent,” “I am satisfied with life,” “So far I have gotten the important things I want in life,” and “If I could live my life over, I would change almost nothing.”

## Results

### Main Analyses

Means, standard deviations (*SD*s), and zero-order correlations between measures are presented in **Table 1**. We ran a series of hierarchical linear regressions in which we entered multiple identities pre-retirement at Step 1 and then multiple identities post-retirement at Step 2 as predictors of satisfaction with retirement, subjective health status, and subjective quality of life.

**Table 1:** Means, standard deviations, and intercorrelations between variables.

Variable	Mean	*SD*	1	2	3	4	5	6	7
(1) Multiple identities pre-retirement	2.99	1.89	-						
(2) Multiple identities post-retirement	3.08	1.88	0.64**	–					
(3) Received social support	5.39	1.38	0.13^†^	0.23**	–				
(4) Provided social support	5.80	1.06	0.14^†^	0.16*	0.75**	–			
(5) Satisfaction with retirement	5.56	1.48	0.16*	0.23**	0.11	0.20**	–		
(6) Subjective health status	5.00	1.54	0.14**	0.30**	0.29**	0.34**	0.30**	–	
(7) Subjective quality of life	5.01	1.34	0.16*	0.30**	0.38**	0.37**	0.48**	0.56**	–

^†^*p* < 0.10, *p < 0.05, **p < 0.01; *N* = 159–171.

#### Satisfaction with Retirement

As shown in **Table 2**, supporting H1a, linear regression analyses indicated a significant effect of multiple identities pre-retirement at Step 1 (β = 0.17, *p* = 0.040) that accounted for 3% of the variance in satisfaction with retirement. When entering multiple identities post-retirement as an additional predictor at Step 2, multiple identities pre-retirement was no longer a significant predictor (β = 0.03, *p* = 0.776) and only multiple identities post-retirement significantly predicted satisfaction in retirement (β = 0.21, *p* = 0.038). This suggests that multiple identities prior to retirement is a significant predictor on its own but it ceases to be a significant predictor once one controls for the shared variance between multiple identities following retirement and retirement satisfaction (supporting H2). Furthermore, it is noteworthy that the variance accounted for by the model that included both predictors (at Step 2) increased significantly to a total of 6%.

**Table 2:** Hierarchical regression analyses: Multiple identities (pre- and post-retirement) predicting satisfaction with retirement, subjective health, and quality of life.

	Step 1					Step 2				
Variable	*b*	*SE*	95% CIs	β	*t*	*b*	*SE*	95% CIs	β	*t*
**Satisfaction with retirement**					
*Intercept*	5.18	0.22	4.74, 5.62	–	23.29**	4.96	0.25	4.47, 5.44	–	20.25**
*Predictors*					
Multiple identities (Pre)	0.13	0.06	0.01,0.25	0.17	2.07	0.02	0.08	–0.14,0.18	0.03	0.77
Multiple identities (Post)						0.17	0.08	0.01,0.34	0.21	2.10*
*R*^2^					0.03*					0.06*
**Subjective health status**					
*Intercept*	4.47	0.23	4.02, 4.92	–	19.70**	4.23	0.25	3.74, 4.72	–	16.96**
*Predictors*					
Multiple identities (Pre)	0.19	0.06	0.07,0.32	0.24	3.02**	0.08	0.08	-0.08,0.24	0.10	0.96
Multiple identities (Post)						0.19	0.08	0.02,0.35	0.22	2.21*
*R*^2^					0.06**					0.09**
**Subjective quality of life**					
*Intercept*	4.76	0.19	4.38, 5.15	–	24.49**	4.54	0.21	4.12, 4.96	–	21.33**
*Predictors*					
Multiple identities (Pre)	0.11	0.06	-0.01,0.21	0.16	1.95^†^	-0.01	0.07	-0.14,0.14	-0.01	-0.01
Multiple identities (Post)						0.17	0.07	0.03,0.32	0.25	2.42*
*R*^2^					0.02^†^					0.06**

^†^*p* < 0.10, *p < 0.05, **p < 0.01; Multiple Identities (Pre) and (Post) refer to multiple identities (number of social group memberships) prior to and following retirement, respectively.

#### Subjective Health Status

Consistent with H1b, multiple identities pre-retirement was a significant predictor of subjective health status at Step 1 (β = 0.24, *p* = 0.003), accounting for 6% of the variance. However, it was not a significant predictor at Step 2 when both multiple identities prior to and following retirement are entered (β = 0.10, *p* = 0.338). Supporting H2, at Step 2 only multiple identities post-retirement was a significant predictor (β = 0.22, *p* = 0.028) and this model accounted for a greater proportion (a total of 9%) of the variance in subjective health status.

#### Subjective Quality of Life

**FIGURE 1:**
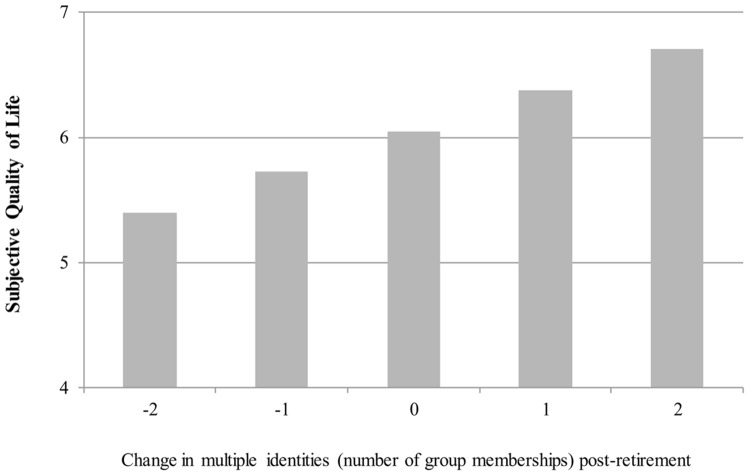
**Change in multiple identities (number of social group memberships) from pre-retirement to post-retirement is associated with subjective quality of life.** Results are presented for a retiree who had three social group memberships prior to retirement.

Supporting H1c, analysis revealed a marginally significant effect of pre-retirement multiple identities at Step 1 (β = 0.16, *p* = 0.053) that explained 2% of the total variance. At Step 2, and consistent with H2, pre-retirement multiple identities were no longer a significant predictor (β = -0.01, *p* = 0.989) but post-retirement multiple identities were (β = 0.25, *p* = 0.017). Together the predictors explained an even greater share (i.e., 6%) of the total variance. In the context of the transition, these results indicate that if a retiree had three group memberships prior to retirement but then lost two group memberships (so that they now had one) their mean subjective quality of life was 5.40 (on a 7-point scale). However, a retiree’s mean subjective quality of life was 6.05 if they maintained three group memberships after retirement and 6.70 if they gained two group memberships (so that they now had five). These results are presented in **Figure 1**.

#### Mediation via Received and Provided Social Support

We examined the extent to which the relationship between multiple identities and satisfaction with retirement was mediated by received and provided social support. For this purpose, we ran bias-corrected bootstrapping multiple mediation analyses with 5000 resamples using PROCESS (Model 4; Hayes, 2013). Multiple mediation analysis has the advantage that it can test the indirect effect through given and provided social support, while simultaneously controlling for the impact of the other variable.

Results indicated that the indirect path from multiple identities post-retirement to satisfaction with retirement through provided social support was significant, γ = 0.04, *SE* = 0.03, 95%CIs [0.01,0.11], while the indirect path through received social support was not significant, γ = -0.01, *SE* = 0.02, 95%CIs [-0.07,0.02], *R*^2^_Model_ = 0.10, Δ*R*^2^_AdditionofMediators_ = 0.03, providing support for H4a (but no support for H3a). These mediation results are displayed in **Figure 1**.

Furthermore, supporting H4b, results for subjective health status showed a significant indirect path from multiple identities post-retirement through provided social support, γ = 0.05, *SE* = 0.03, 95%CIs [0.01,0.13]. However, they revealed a non-significant indirect path through received social support, γ = 0.01, *SE* = 0.02, 95%CIs [-0.03,0.07], *R*^2^_Model_ = 0.19, Δ*R*^2^_AdditionofMediators_ = 0.09, providing no support for H3b.

**FIGURE 2:**
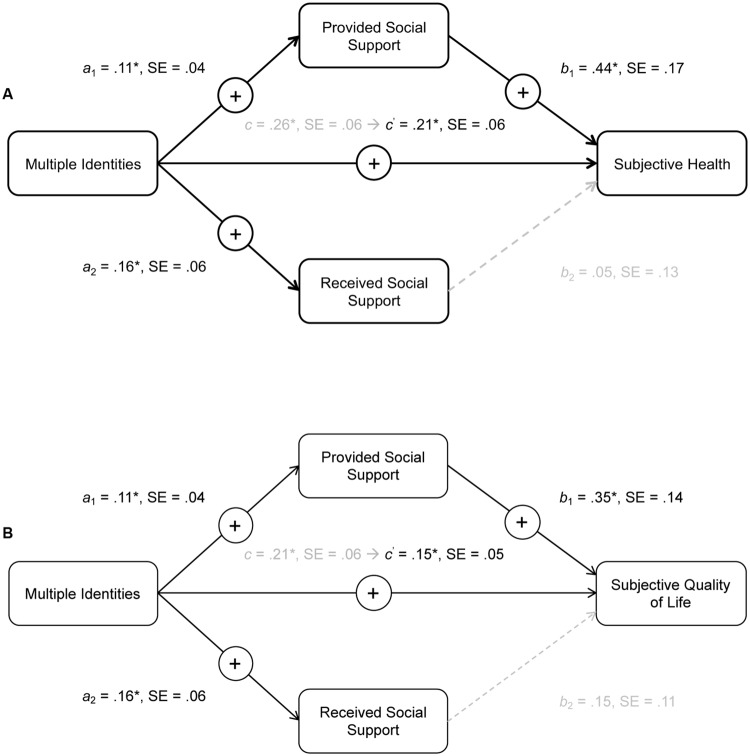
**Mediation displaying unstandardized coefficients for the direct and indirect paths from multiple identities (number of social group memberships) through provided and received social support to **(A)** satisfaction with retirement and **(B)** subjective quality of life.** Asterisks indicate statistically significant coefficients (*p < 0.05).

Finally, and as shown in **Figure 2**, results supported H4c by showing that the indirect path from multiple identities post-retirement to quality of life through provided social support was significant, γ = 0.05, *SE* = 0.03, 95%CIs [0.01,0.13]. At the same time, there was no support for H3c in so far as the indirect path through received social support was non-significant, γ = 0.01, *SE* = 0.02, 95%CIs [-0.03,0.07], *R*^2^_Model_ = 0.23, Δ*R*^2^_AdditionofMediators_ = 0.14. Consistent with H4, these mediation analyses thus indicate that the relationship between multiple identities and health and well-being outcomes was not mediated by retirees’ receipt of support but was mediated by their provision of it to others.

In addition, we examined whether received social support was a significant mediator when examined on its own (without controlling simultaneously for provided social support). This analysis indicated that the indirect path from multiple identities post-retirement to satisfaction with retirement through received social support was significant, γ = 0.02, *SE* = 0.02, 95%CIs [0.00,0.07], *R*^2^_Model_ = 0.08, Δ*R*^2^_AdditionofMediator_ = 0.02. Furthermore, results revealed that the indirect effect of multiple identities post-retirement through received social support was also significant in the analysis for subjective health status, γ = 0.05, *SE* = 0.02, 95%CIs [0.01,0.10], *R*^2^_Model_ = 0.13, Δ*R*^2^_AdditionofMediator_ = 0.05, and subjective quality of life, γ = 0.06, *SE* = 0.03, 95%CIs [0.02,0.13], *R*^2^_Model_ = 0.20, Δ*R*^2^_AdditionofMediator_ = 0.11. In sum, results indicate that, consistent with previous research, received social support is a significant mediator of the link between multiple identities and health when examined on its own. In addition, results also indicate that in the present sample the link between multiple identities and health through received social support is accounted for by the shared variance with provided social support.

### Sensitivity Analyses

We conducted four further tests to establish the robustness of the patterns observed above. First, it is possible that the effects of possessing more social identities might be linear but also that they saturate such that once a person has many rather than few groups any additional group might provide fewer health benefits. To address this question, we examined whether there was evidence of a curvilinear relationship between multiple identities and dependent variables in order to examine whether the benefits of each additional social group membership decreases as the number of social group memberships increases. This involved computing the quadratic term of multiple identities following retirement (to reduce collinearity between predictors, multiple identities were *Z*-standardized prior to calculating the quadratic term) and then running a series of linear regression analyses in which the (*Z*-standardized) linear term was added at Step 1 and the quadratic term was added as a predictor at Step 2. Analysis revealed that the quadratic term was unrelated to, and did not account for additional variance beyond that accounted for by the linear term in the prediction of, satisfaction with retirement, *b* = -0.03, 95%CIs [-0.26,0.20], β = -0.02, Δ*R*^2^ < 0.001, *p* = 0.780, subjective health status, *b* = -0.21, 95%CIs [-0.45,0.02], β = -0.13, Δ*R*^2^ = 0.018, *p* = 0.075, and subjective quality of life, *b* = -0.10, 95%CIs [-0.30,0.11], β = -0.72, Δ*R*^2^ = 0.005, *p* = 0.364. In sum, there was no evidence of a curvilinear relationship between multiple identities and dependent variables, suggesting that there were no diminishing marginal returns associated with multiple identities and that the relationships never become negative.

Second, to gain insight into the directionality of the relationships in our focal mediation model, we ran additional sensitivity analyses examining the reversed mediation from better health via social support to multiple identities. Results indicated that none of the reversed indirect paths from health measures (as independent variables) via received and provided social support to multiple identities post-retirement (as the dependent variable) were significant. More specifically, the (reversed) indirect paths to multiple identities post-retirement through both received and provided social support were not significant when examining (a) satisfaction with retirement as the independent variable (received support: γ = 0.03, *SE* = 0.03, 95%CIs [-0.01,0.12]; provided support: γ = 0.01, *SE* = 0.03, 95%CIs [-0.06,0.08], *R*^2^_Model_ = 0.10, Δ*R*^2^_AdditionofMediators_ = 0.03), (b) subjective health status as the independent variable (received support: γ = 0.05, *SE* = 0.05, 95%CIs [-0.02,0.16]; provided support: γ = -0.01, *SE* = 0.05, 95%CIs [-0.11,0.10], *R*^2^_Model_ = 0.11, Δ*R*^2^_AdditionofMediators_ = 0.02), or (c) subjective quality of life as the independent variable (received support: γ = 0.07, *SE* = 0.07, 95%CIs [-0.05,0.21]; provided support: γ = 0.01, *SE* = 0.07, 95%CIs [-0.12,0.15], *R*^2^_Model_ = 0.10, Δ*R*^2^_AdditionofMediators_ = 0.02).

Finally, we ran additional analyses that included the number of highly important groups (rated above the mid-point of the scale — i.e., 5 or higher on the 1 to 7 scale). Results indicated that the number of highly important identities post-retirement (while controlling for those pre-retirement) were positively associated with all health outcomes (predicting a similar or a slightly greater proportion of the variance across the variables): (a) satisfaction with retirement, β = 0.22, *p* = 0.022, *R*^2^_Model_ = 0.05, (b) subjective health status, β = 0.34, *p* < 0.001, *R*^2^_Model_ = 0.13, and (c) subjective quality of life, β = 0.27, *p* = 0.004, *R*^2^_Model_ = 0.08. This confirms the point that it is people’s sense of psychological connectedness with groups that determines health outcomes.

## Discussion

In the present research, we examined the extent to which retirees’ multiple identities — both before and after the transition to retirement — are associated with their adjustment to retirement and their health. Results show that the more social group memberships retirees had following retirement, (a) the more satisfied they were with retirement, and (b) the better their subjective health and (c) the better their subjective quality of life (supporting H1). Furthermore, evidence indicated that these health benefits primarily derived from multiple identities following (but not prior to) retirement (supporting H2). The present findings demonstrate that being less well connected before retirement need not be detrimental as people can still experience good health in retirement to the extent that they develop and build multiple social identities.

Sensitivity analyses also indicated that there was no evidence of a curvilinear relationship between multiple identities and retirement satisfaction and health, suggesting that there were no diminishing marginal returns of multiple identities and, more particularly, that the relationships never become negative. Finally, speaking to the mechanism through which multiple identities protect health and well-being in retirement, results revealed an indirect effect through giving social support but not through receiving social support (when controlling simultaneously for giving social support). Specifically, retirees who had multiple identities indicated that they provided more social support to others and this in turn was associated with greater satisfaction with retirement, better health, and greater subjective quality of life (providing support for H4).

The present findings make at least three important contributions to the literatures on retirement, multiple identities, and health. First, we are only just starting to understand how social group memberships are implicated in people’s ability to adjust to a life change that most individuals experience at some point in their lifetime — namely, retirement (Steffens et al., 2016a). Yet this prior research provided limited insight into the extent to which multiple identities impact retirees’ adjustment and health because the research (a) relied on groups whose specification was fixed and pre-determined, and (b) did not consider retirement satisfaction (as a direct indicator of retirees’ psychological adjustment to retirement). Our results extend this research and provide more conclusive support for the idea that individuals’ *multiple identities* — subjective group memberships that they *self-identify* as being part of who they are — are associated with successful retirement. Retirees who felt connected to a greater number of social groups not only had greater subjective quality of life but also were more satisfied with retirement and experienced better health.

Second, our results shed some light on the processes through which multiple identities are associated with better health in retirement. Previous research that explored these issues has shown that social identities are health-enhancing because they provide people with a sense that they are supported by other people (Haslam et al., 2005, 2016b; Crabtree et al., 2010; Avanzi et al., 2015; Walter et al., 2016). However, the present study extends our understanding of the various ways in which multiple identities are associated with health. Specifically, by examining the provision and receipt of social support simultaneously, our findings indicate that retirees’ multiple identities indirectly impacted their satisfaction with retirement and health primarily because these are a basis for providing social support. This is not to say that receiving social support is unimportant for health and well-being. Indeed, if one does not control for provided social support, received social support is a significant mediator. Nevertheless, in line with previous research (Brown et al., 2003; Abolfathi Momtaz et al., 2014), it appears that, when examined *simultaneously*, the provision of social support explains more variance in health than its receipt, and that provision of social support is a particularly powerful mechanism in the multiple identities–health link. More generally, then, this suggests that in life-changing contexts such as retirement, where individuals are stripped of one important way to contribute to society, multiple identities provide a vehicle for continuing to make a significant contribution to the lives of others and thereby to sustain a healthy life.

Third, the present results suggest that the process of retirement might be enhanced through facilitation of retirees’ social group-based connections to others. Thus, in addition to making financial, residential, medical care, and physical activity plans, our findings suggest that it is also important for retirees to plan how they might stay (or become) socially connected to groups that can provide them with a sense of communality and belonging. Moreover, the fact that beneficial effects were strongest for retirees who had a greater number of social identities post-retirement suggests that these plans need not be constrained by a person’s social circumstances prior to retirement. For, as the present data show, building one’s network of social group memberships after retirement can bring health and well-being gain. While this network may be more difficult to develop and embed for those less well connected before they retire, there is evidence that they can be strengthened by social interventions that explicitly target social disconnection (e.g., GROUPS 4 HEALTH; Haslam et al., 2016a). There is clearly mileage in future research seeking to establish whether such an intervention — whose goal is explicitly to build and maintain multiple social identities — can be successfully adapted to promote the health and well-being of retirees.

### Limitations and Future Research

The present research is not without limitations. Primary amongst these is the fact that, because the study has a cross-sectional design it cannot establish causality in the associations investigated here. Future research should therefore employ longitudinal and experimental (intervention) studies in order to shed light on directionality (as do Steffens et al., 2016a). In this regard it is worth noting, though, that we do not rule out the possibility that health may also increase people’s willingness to provide social support — even though in the present study there was no evidence of this reversed mediation. Indeed, research on the link between giving and happiness shows that this link is likely to be mutually reinforcing (bidirectional) such that giving increases happiness, which in turn, increases giving (Thoits and Hewitt, 2001; Aknin et al., 2012). Nevertheless, it would clearly be useful for long-term panel studies to shed light on this issue by exploring the strength of these different pathways. In this regard too, we cannot exclude the possibility that part of the variance may be explained by individual differences (for example, people who are cognitively more flexible or more open to experience may be more likely to join new groups in retirement). Having said this, research from other fields shows that effects of multiple group memberships are not reducible to stable individual differences. In this regard, an experimental study by Jones and Jetten (2011) showed that reflecting on many, rather than few, group memberships led to subsequent increases in resilience, while an intervention study by Haslam et al. (2016a) that focused on the maintenance and development of social group memberships produced improvement in participants’ health. Furthermore, Steffens et al. (2016b) controlled for the Big Five in their studies that examined the link between multiple social identities and creativity and found that results could not be reduced to differences in personality. Nevertheless, it is possible that retirees’ willingness and capacity to develop new multiple identities may in part be impacted by other variables, a possibility that future research should examine.

Moreover, it is certainly the case that one’s social life involves not only group-based social ties but also those with significant individuals, which have been shown to be important in adjusting to retirement. In this context, spousal relationships have been highlighted as particularly important (Kupperbusch et al., 2003; Davey and Szinovacz, 2004; Bushfield et al., 2008), but as these were not the focus of the present research, we cannot comment on the contribution that (particular) individual relationships make relative to group-based relationships. Interestingly, though, relationships that people have with significant others tend not to be confined to one-on-one interaction, but often occur in the context of wider group-based interaction (with the family in the case of a spouse, or a work team in the case of a professional colleague). While we are not discounting the important role that one-on-one relationships have for health and well-being, there is a growing body of evidence from a variety of (healthy, vulnerable, and clinical) populations which indicates that social connectedness to *groups* is a unique and reliable predictor of several health outcomes (for reviews, see Cruwys et al., 2014; Jetten et al., 2014; Steffens et al., 2016c). Along these lines, Sani et al. (2012) have shown that health effects derive from feeling oneself to be part of a social group and not merely from one’s amount of social contact. Furthermore, Haslam et al. (2014) have shown that it is group ties and not one-on-one ties that predict subsequent cognitive health among older adults, and Glei et al. (2005) have found that group-based activities are more important than individual engagement with significant others (with a spouse, close relative, friend) in protecting cognitive health over time. Having said this, further research is needed to disentangle and quantify the extent to which retirees’ health benefits derive from different aspects of their social group memberships as well as other aspects of their social life. For instance, future research should examine the extent to which adjustment to retirement is impacted by the perceived compatibility between the multiple groups that retirees are part of (Iyer et al., 2009) as well as the sense of continuity in their group memberships and associated sense of self (Sani, 2008; Sani et al., 2008).

Furthermore, there would also be value in future work that expands the scope of the potential outcomes that flow from having multiple identities. For example, it would be interesting to investigate not only retirees’ sustained mental and physical health but also other aspects of an engaged healthy life such as cognitive function (e.g., memory and creativity; Haslam et al., 2014; Steffens et al., 2016b) and resilience (e.g., the capacity to bounce back from significant set-backs; Jones et al., 2011; Cruwys et al., 2013). Similarly, it would be worthwhile disentangling the active ingredients in the provision of social support that are associated with better health and well-being. In this regard, it is noteworthy that the association between social support and the various health outcomes was overall of moderate magnitude, while the association between multiple identities and both forms of social support was only in the weak to moderate range (*r*s = 0.16 and 0.23). To provide a better understanding of these relationships, future research might shed light on the extent to which (a) multiple identities are related to different aspects of provision of support, such as one’s motivation, actual supportive behaviors, or the impact that giving has on others (Bolino and Grant, 2016) and (b) these different elements have benefits for people’s health and well-being.

Finally, in the present research we focused on subjectively experienced (and reported) support and health (rather than objective indicators of support and health) and future research might extend the present examination by including other more objective indicators of these measures (see also Fernández-Ballesteros, 2011). It is important to note though, that people are generally capable of reporting meaningfully the way they experience their health and reviews indicate that reported quality of life and subjective health status are powerful predictors of a range of other third-party rated and objective health measures (including mortality; Pavot et al., 1991; Idler and Benyamini, 1997). Accordingly, while previous research might lead one to expect weaker effects for objective physical than subjective psychological health measures (e.g., see Steffens et al., 2016c), there are no grounds for expecting the pattern of findings to be substantively different from that observed in the present research.

## Conclusion

The present research investigated how multiple identities in the transition to retirement are associated with successful adjustment, as well as health and well-being. Our results show that multiple identities following retirement are associated not only with greater satisfaction with retirement but also with better subjective health and enhanced subjective quality of life. Furthermore, the study is the first to demonstrate that the health-protective benefits of having multiple identities arise in part from the fact that they give people more opportunity to contribute to the lives of others through the provision of social support. This observation is important for theoretical reasons but also has the potential to inform practical interventions — in pointing to the importance of cultivating multiple social identities among retirees with a view to fostering their resilience.

Together, these various findings provide a new dimension to our understanding of the importance of networks of social group memberships in retirement. In particular, they suggest that, in the process of giving up membership in work-related group(s), access to multiple social identities allows people to continue to be active members of other valued communities and thereby to continue to have meaningful and healthy lives. Indeed, in ways that would surely please John F. Kennedy, we can see that, in retirement, multiple group memberships provide a basis for people to reap the benefits not of what others can do for them but of what they can do for others.

## Author Contributions

NS, JJ, CH, TC, and AH developed the study concept and designed the research. NS performed the statistical analyses. NS drafted the manuscript. All authors edited the manuscript. All authors read and approved the final manuscript.

## Conflict of Interest Statement

The authors declare that the research was conducted in the absence of any commercial or financial relationships that could be construed as a potential conflict of interest.
